# Supplementary material for: Different Geographic Strains of Dinoflagellate Karlodinium veneficum Host Highly Diverse Fungal Community and Potentially Serve as Possible Niche for Colonization of Fungal Endophytes
Source: Int J Mol Sci. 2023 Jan 14;24(2):1672. doi: 10.3390/ijms24021672 (PMC9865425; doi:10.3390/ijms24021672)
Supplement: Supplementary file 1 [file ijms-24-01672-s001.zip › Supplementary Figures.pdf]

Supplementary Figures

Supplementary Figure S1. Rarefaction curves of the 13 samples.

Supplementary Figure S2. Heatmap analysis of the whole fungal communities living with *Karlodinium veneficum* at class level.

Supplementary Figure S3. Phylogenetic tree derived from Maximum Likelihood analysis based on ITS sequences of representative fungal ASVs feature sequences obtained in this study.

Supplementary Figure S4. The phylogenetic relationships of the 13 *Karlodinium veneficum* investigated in this work.

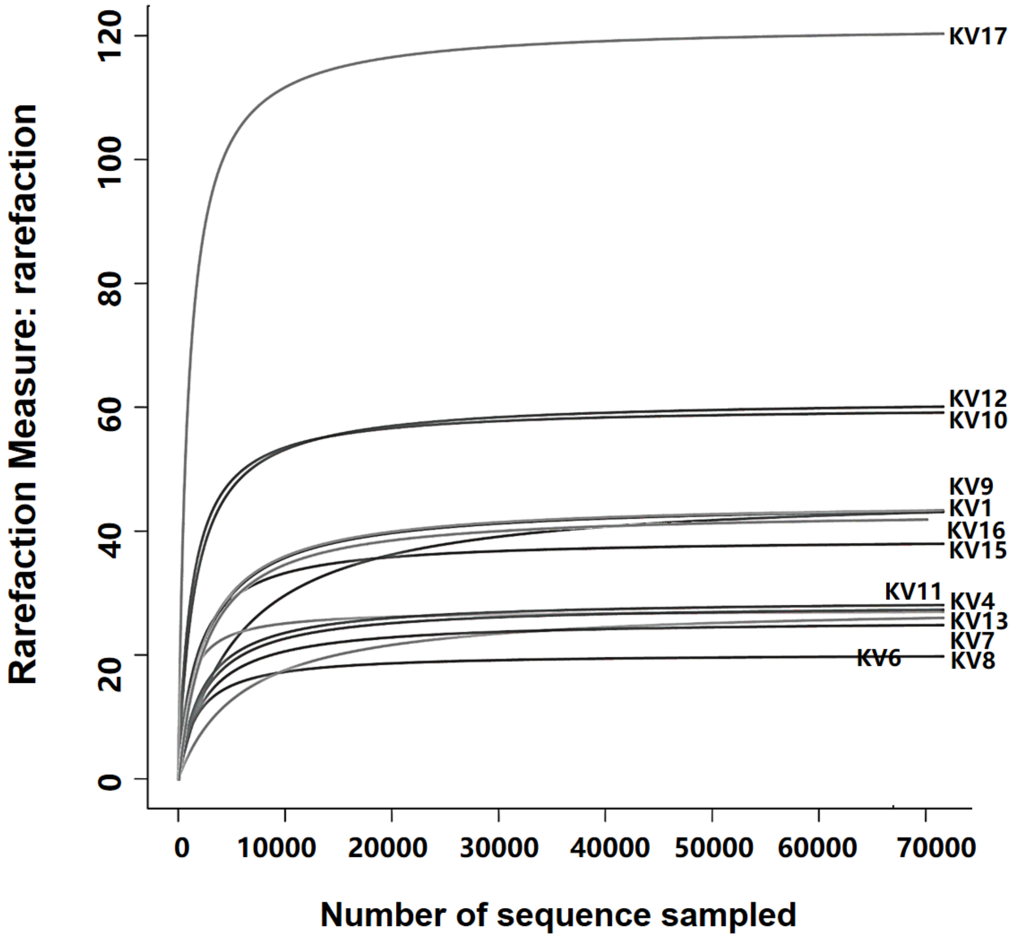

Supplementary Figure S1. Rarefaction curves of the 13 samples.

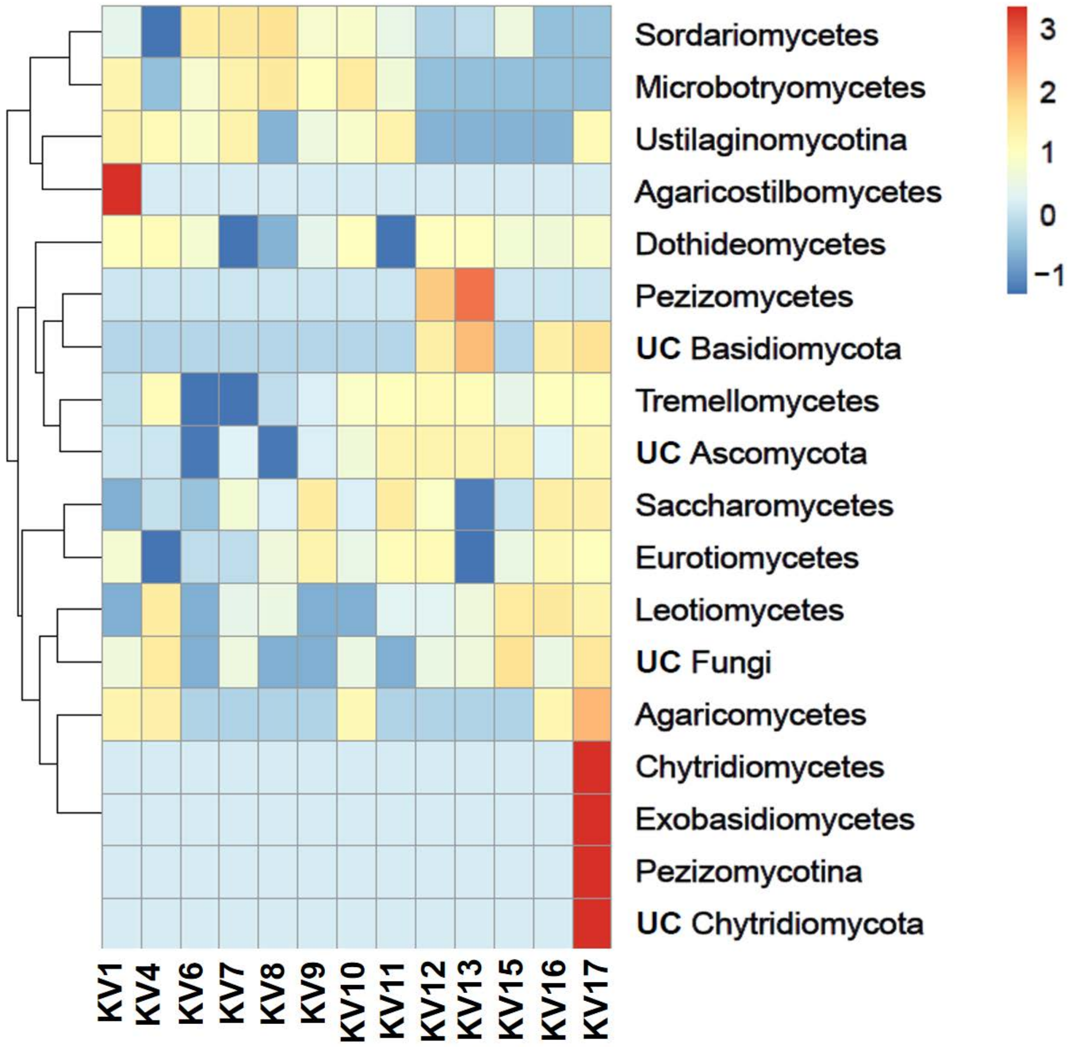

Supplementary Figure S2. Heatmap analysis of the whole fungal communities living with *Karlodinium veneficum* at class level. UC: unclassified.

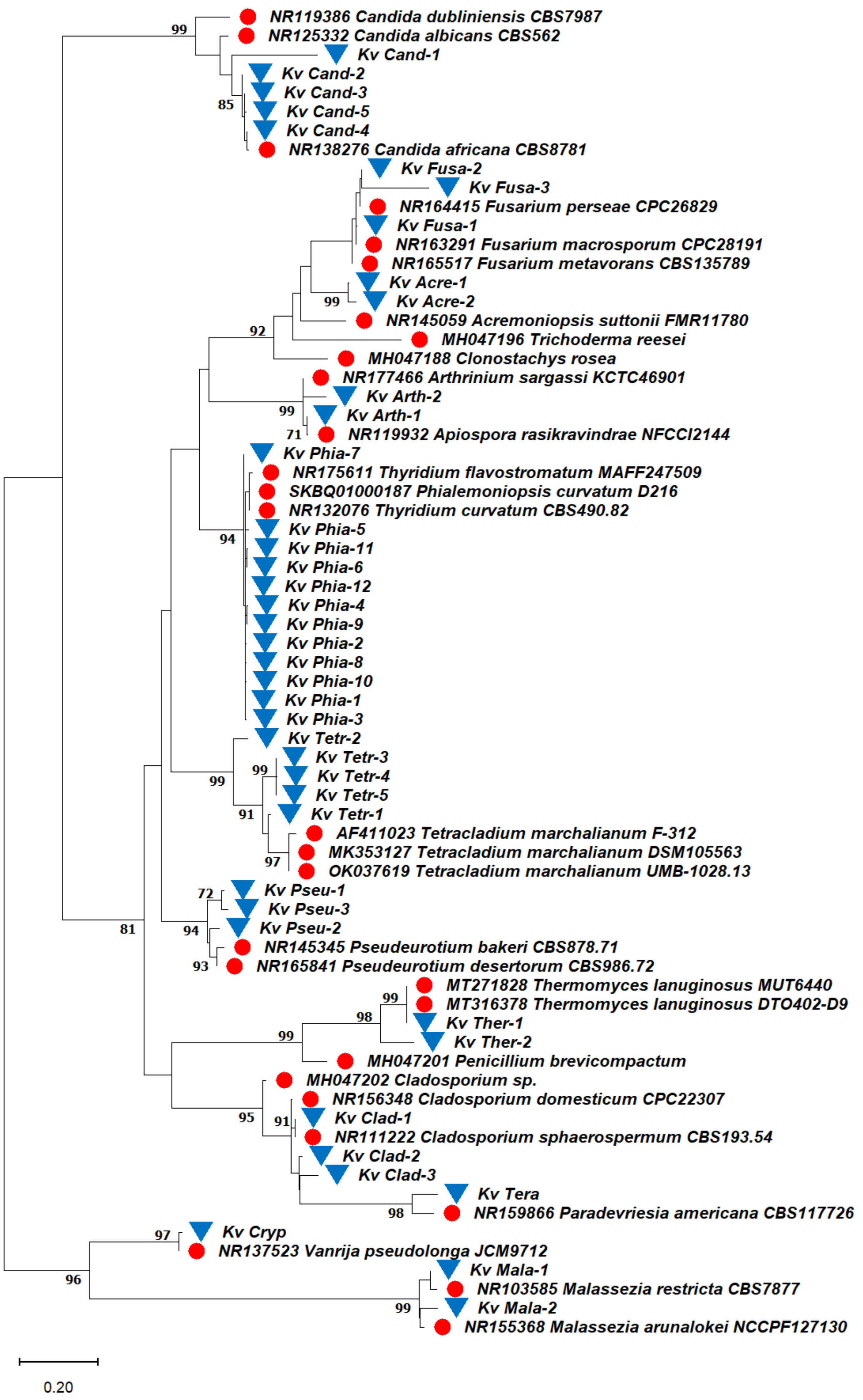
NR119386 Candida dubliniensis CBS7987
NR125332 Candida albicans CBS562
Kv Cand-1
Kv Cand-2
Kv Cand-3
Kv Cand-5
Kv Cand-4
NR138276 Candida africana CBS8781
Kv Fusa-2
Kv Fusa-3
NR164415 Fusarium perseae CPC26829
Kv Fusa-1
NR163291 Fusarium macrosporum CPC28191
NR165517 Fusarium metavorans CBS135789
Kv Acre-1
Kv Acre-2
NR145059 Acremoniopsis suttonii FMR11780
MH047196 Trichoderma reesei
MH047188 Clonostachys rosea
NR177466 Arthrinium sargassi KCTC46901
Kv Arth-2
Kv Arth-1
NR119932 Apiospora rasikravindrae NFCCI2144
Kv Phia-7
NR175611 Thyridium flavostromatum MAFF247509
SKBQ01000187 Phialemoniopsis curvatum D216
NR132076 Thyridium curvatum CBS490.82
Kv Phia-5
Kv Phia-11
Kv Phia-6
Kv Phia-12
Kv Phia-4
Kv Phia-9
Kv Phia-2
Kv Phia-8
Kv Phia-10
Kv Phia-1
Kv Phia-3
Kv Tetr-2
Kv Tetr-3
Kv Tetr-4
Kv Tetr-5
Kv Tetr-1
AF411023 Tetracladium marchalianum F-312
MK353127 Tetracladium marchalianum DSM105563
OK037619 Tetracladium marchalianum UMB-1028.13
Kv Pseu-1
Kv Pseu-3
Kv Pseu-2
NR145345 Pseudeurotium bakeri CBS878.71
NR165841 Pseudeurotium desertorum CBS986.72
MT271828 Thermomyces lanuginosus MUT6440
MT316378 Thermomyces lanuginosus DTO402-D9
Kv Ther-1
Kv Ther-2
MH047201 Penicillium brevicompactum
MH047202 Cladosporium sp.
NR156348 Cladosporium domesticum CPC22307
Kv Clad-1
NR111222 Cladosporium sphaerospermum CBS193.54
Kv Clad-2
Kv Clad-3
Kv Tera
NR159866 Paradevriesia americana CBS117726
Kv Cryp
NR137523 Vanrija pseudolonga JCM9712
Kv Mala-1
NR103585 Malassezia restricta CBS7877
Kv Mala-2
NR155368 Malassezia arunalokei NCCPF127130
99
85
99
92
99
71
94
99
99
91
97
72
94
93
81
99
98
99
95
91
98
97
96
99
0.20

Supplementary Figure S3. Phylogenetic tree derived from Maximum Likelihood analysis based on 208-bp of 5.8S-ITS2 DNA sequence data. Analysis was performed in MEGA X (Kumar et al., 2018), using General Time Reversible (Nei and Kumar, 2000) and gamma-distribution rate variation (GTR+G model) with 1000 bootstrap replicates. The analysis involved 70 nucleotide sequences, including representative fungal ASVs feature sequences obtained in this study (● with Kv prefix), and the reference fungal strain sequences retrieved from GenBank (▼ with accession number). ML bootstrap support (BS) values are shown at the nodes (BS > 70 %). The tree is drawn to scale, with branch lengths measured in the number of substitutions per site. The scale bar indicates the nucleotide substitutions per position.

References;

Kumar, S.; Stecher, G.; Li, M.; Knyaz, C.; Tamura, K. MEGA X: Molecular evolutionary genetics analysis across computing platforms. *Molecular Biology and Evolution* **2018**, *35*, 1547-1549.

Nei, M.; Kumar, S. Molecular Evolution and Phylogenetics. **2000**, Oxford University Press, New York.

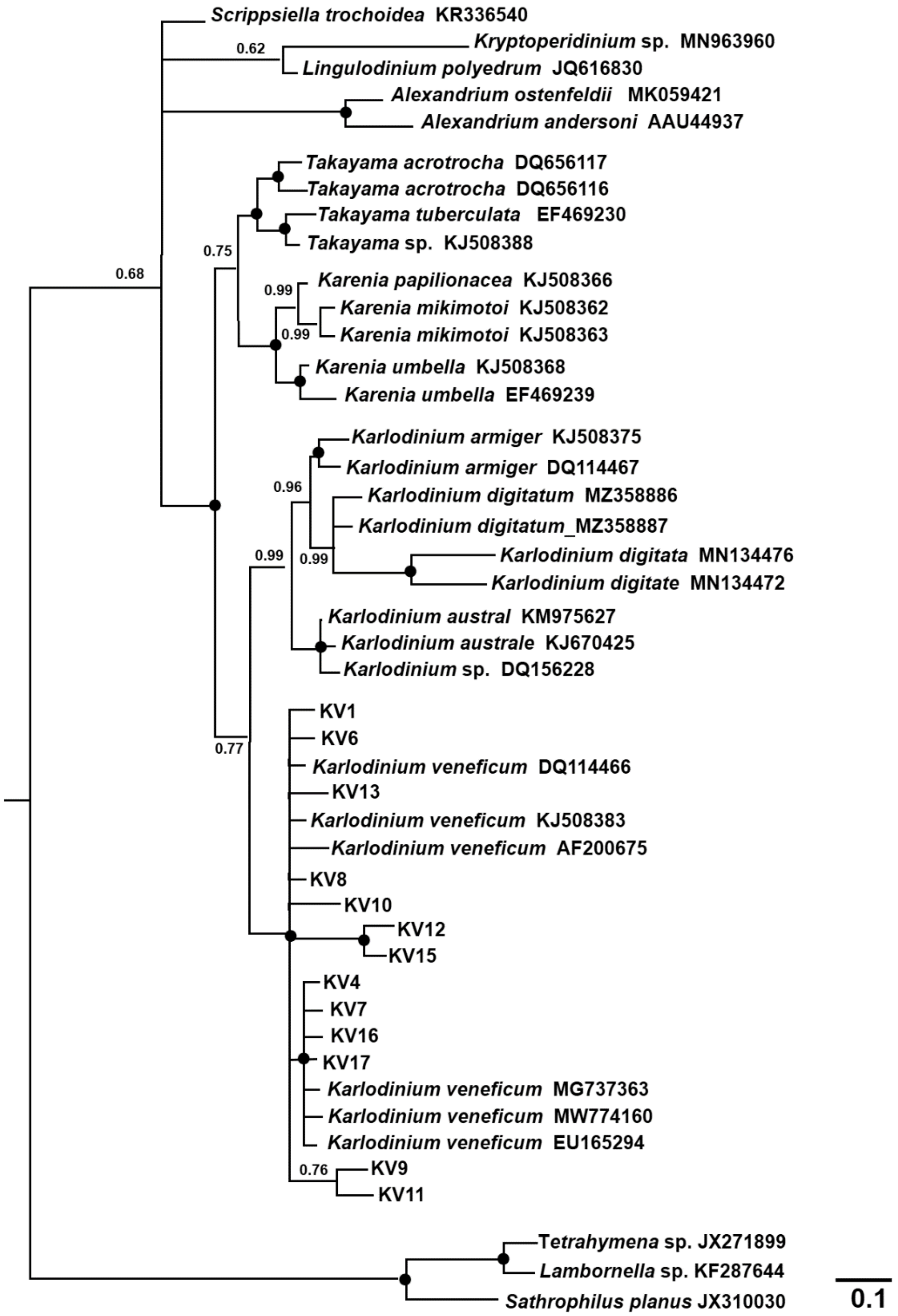

Supplementary Figure S4. The phylogenetic relationships of the 13 *Karlodinium veneficum* investigated in this work. Phylogenetic reconstruction was inferred from partial LSU rRNA gene sequences using the Bayesian-Inference (BI) method as described in Deng et al. (2019, 2021). Numbers at the nodes represent BI posterior probabilities. Black circles (●) indicate maximal support (1.00). GenBank accession number of each sequence is noted following the species name. All branches are drawn to scale.

References;

Deng, Y.Y.; Li, F.T.; Hu, Z.X.; Yue, C.X.; Tang, Y.Z. The implication inferred from the expression of small heat-shock protein genes in dinoflagellate resting cysts buried in marine sediment. *Diversity* **2021**, *13*, 471.

Deng, Y.Y., Hu, Z.X., Chai, Z.Y., Tang, Y.Z. Cloning and comparative studies of proliferating cell nuclear antigen (PCNA) genes for nine dinoflagellates. *Journal of Applied Phycology* **2019**, *31*, 2969-2979.
