# Supplementary material for: Different Geographic Strains of Dinoflagellate Karlodinium veneficum Host Highly Diverse Fungal Community and Potentially Serve as Possible Niche for Colonization of Fungal Endophytes
Source: Int J Mol Sci. 2023 Jan 14;24(2):1672. doi: 10.3390/ijms24021672 (PMC9865425; doi:10.3390/ijms24021672)
Supplement: Supplementary file 1 [file ijms-24-01672-s001.zip › Supplementary Table S5.pdf]

Table S5. Previous records of 15 abundant genera from marine habitats (This table should not be considered exhaustive, papers included in this table only represent parts of recent studies reported these genera from marine habitats).

Genus	Abundances[1]	Occurrence in marine habitat[2]	Common in marine habitat[3]	References
Thyridium[4]	36.40%	√		Li, 2013; Li et al., 2016; Xu et al., 2017; Mahanty et al., 2019; Maamar et al., 2020; Taha et al., 2021; Wang et al., 2021
Pseudeurotium	11.54%	√		Gao et al., 2008; Li, 2013; Henríquez et al. 2014; Li et al., 2016; Duarte et al., 2018; da Silva et al., 2020; Wang et al., 2021
Candida	8.23%	√	√	Reviewed in Richards et al., 2012, Raghukumar et al., 2017, Grossart et al. 2019, Sen et al. 2022; Kutty and Philip, 2008; Singh et al., 2010; Edgcomb et al., 2011; Fell, 2012; Li, 2013; Orsi et al., 2013; Taylor and Cunliffe, 2014; Zhang et al., 2014; Jones et al., 2015; Li et al. 2016; Xu et al., 2016; Hassett et al., 2020; Sen et al., 2021; Wang et al., 2021
Cladosporium	3.89%	√	√	Reviewed in Richards et al., 2012, Raghukumar et al., 2017, Grossart et al. 2019, Sen et al. 2022 Höller et al. 2000; Zhdanova et al., 2000; Damare et al., 2006; Singh et al., 2010; Li et al., 2014; Rédou et al., 2015; Zhang et al., 2015; Vera et al., 2017; Xu et al., 2017; Fotedar et al., 2018; Maamar et al., 2020; Pham et al., 2021; Sen et al., 2021; Wang et al., 2021
UC Ascomycota	3.89%	-	-	-

Arthrinium	3.31%	√	√	Reviewed in Raghukumar et al., 2017 and Sen et al. 2022; Höller et al. 2000; Miao et al., 2006; Burgaud et al., 2013; Li, 2013; Li et al., 2014; Wang et al., 2015; Venkatachalam et al., 2015; Li et al., 2016; Vera et al., 2017; Kwon et al., 2021; Pham et al., 2021; Wang et al., 2021
Tetracladium	2.59%	√		Rämä et al., 2014; Kalenitchenko et al., 2015; Chauvet et al., 2016; Li et al., 2016; Wang et al., 2021
Cryptococcus	2.54%	√	√	Reviewed in Richards et al., 2012; Raghukumar et al., 2017, Grossart et al. 2019, Sen et al. 2022; Takishita et al., 2006; Kutty and Philip, 2008; Edgcomb et al., 2011; Fell, 2012; Orsi et al., 2013; Li et al., 2014; Rédou et al., 2014; Jones et al. 2015; Li et al., 2016; Xu et al., 2016
Malassezia	2.17%	√	√	Reviewed in Richards et al., 2012, Raghukumar et al., 2017, Grossart et al. 2019, Sen et al. 2022; Nakagaki et al., 2000; Kutty and Philip, 2008; Edgcomb et al., 2011; Orsi et al., 2013; Amend 2014; Li et al. 2016; Xu et al., 2016; Lai et al., 2017; Sen et al., 2021
Fusarium	1.95%	√	√	Reviewed in Richards et al., 2012, Raghukumar et al., 2017, Jones et al., 2019, Grossart et al. 2019. Bugni and Ireland 2004; Damare et al., 2006; Li and Wang, 2009; Singh et al., 2010; Passarini et al., 2011; Nasrawi 2012; Steliga et al., 2012; Rédou et al., 2015; Zhang et al., 2015; Fuentes et al., 2016; Li et al., 2016; Sen et al., 2021; Wang et al., 2021
Acremonium	1.79%	√	√	Reviewed in Raghukumar et al., 2017, Sen et al. 2022; Höller et al. 2000; Zhdanova et al., 2000; Zuccaro et al., 2004; Panno et al., 2011; Li, 2013; Rédou et al., 2015; Venkatachalam et al., 2015; Li et al. 2016; Silber et al., 2016; Maamar et al., 2020; Wang et al., 2021

UC Fungi	1.40%	-	-	-
Phialocephala	1.18%	√		Li et al., 2007; Chen et al., 2010; Ebel, 2012; Li et al., 2016; Zhang et al., 2018; Martorell et al., 2021; Wang et al., 2021
Teratosphaeria	1.09%	√		Sakayaroj et al., 2010; Li et al., 2016; Subrmaniyan et al., 2018; Neethu et al., 2019; Wang et al., 2021
Thermomyces	1.08%	√		Li et al., 2016; Sobolevskaya et al., 2021; Wang et al., 2021

UC: unclassified.

[1] **Abundances: Represents relative abundances of the genus in the entire fungal community in the present study.**

[2] **The genus has been previously reported from marine habitats.**

[3] **The genus has been reported to be common in marine environments.**

[4] ***Thyridium*: formerly as *Phialemoniopsis* (**Sugita and Tanaka, 2022) **and *Phialemonium*** (Perdomo et al. 2013).

Sugita, R.; Tanaka, K. *Thyridium* revised: Synonymisation of *Phialemoniopsis* under *Thyridium* and establishment of a new order, Thyridiales. *MycoKeys* **2022**, *86*, 147.

Perdomo, H;; Garcia, D.; Gene, J.; Cano, J.; Sutton, D.A.; Summerbell, R.; Guarro, J. *Phialemoniopsis*, a new genus of Sordariomycetes, and new species of *Phialemonium* and *Lecythophora*. *Mycologia* **2013**, *105*, 398-421.

References:

Amend, A. From dandruff to deep-sea vents: *Malassezia*-like fungi are ecologically hyper-diverse. *PLoS Path.* **2014**, *10*, e1004277.

Al-Nasrawi, H.M. Biodegradation of crude oil by fungi isolated from gulf of Mexico. *J Bioremediat. Biodegrad.* **2012**, *3*, 147-152.

Burgaud, G.; Woehlke, S.; Rédou, V.; Orsi, W.; Beaudoin, D.; Barbier, G.; Biddle, J.F.; Edgcomb, V.P. Deciphering the presence and activity of fungal communities in marine sediments using a model estuarine system. *Aquat. Microb. Ecol.* **2013**, *70*, 45-62.

Bugni, T.S.; Ireland, C.M. Marine-derived fungi: a chemically and biologically diverse group of microorganisms. *Nat. Prod. Rep.* **2004**, *21*, 143-163.

Chen, L.; Li, D.H.; Cai, S.X.; Wang, F.P.; Xiao, X.; Gu, Q.Q. A new cytotoxic metabolite from a deep sea derived fungus, *Phialocephala* sp. *Acta Pharm. Sin*. **2010**, *45*, 1275-1278.

Chauvet, E.; Cornut, J.; Sridhar, K.R.; Selosse, M.-A.; Baerlocher, F. Beyond the water column: aquatic hyphomycetes outside their preferred habitat. *Fungal Ecol.* **2016**, *19*, 112-127.

da Silva, T.H.; Silva, D.A.S.; de Oliveira, F.S.; Schaefer, C.E.G.R.; Rosa, C.A.; Rosa, L.H. Diversity, distribution, and ecology of viable fungi in permafrost and active layer of Maritime Antarctica. *Extremophiles* **2020**, *24*, 565-576.

Damare, S.; Raghukumar, C.; Raghukumar, S. Fungi in deep-sea sediments of the Central Indian Basin. *Deep Sea Res. Part I: Oceanogr. Res. Pap.* **2006**, *53*, 14-27.

Duarte, A.W.F.; Barato, M.B.; Nobre, F.S.; Polezel, D.A.; de Oliveira, T.B.; dos Santos, J.A.; Rodrigues, A.; Sette, L.D. Production of cold-adapted enzymes by filamentous fungi from King George Island, Antarctica. *Polar Biol.* **2018**, *41*, 2511-2521.

Edgcomb, V.P.; Beaudoin, D.; Gast, R.; Biddle, J.F.; Teske, A. Marine subsurface eukaryotes: the fungal majority. *Environ. Microbiol.* **2011**, *13*, 172-183.

Ebel, R. In *Marine fungi and fungal-like organisms.*, Jones EBG, KL, P., Eds.; Natural products from marine-derived fungi; Walter de Gruyter GmbH & Co KG: Berlin, **2012**.

Fell, J. In *Marine Fungi and Fungal-like Organisms*, Jones EBG, KL, P., Eds.; Yeasts in marine environments; Walter de Gruyter GmbH & Co KG: Berlin/Boston, **2012**; pp. 91-102.

Fuentes, M.E.; Quiñones, R.A. Carbon utilization profile of the filamentous fungal species *Fusarium fujikuroi*, *Penicillium decumbens*, and *Sarocladium strictum* isolated from marine coastal environments. *Mycologia* **2016**, *108*, 1069-1081.

Fotedar, R.; Kolecka, A.; Boekhout, T.; Fell Jack,W.; Al-Malki, A.; Zeyara, A.; Al Marri, M. Fungal diversity of the hypersaline Inland Sea in Qatar. *Bot. Mar.* **2018**, *61*, 595.

Gao, Z.; Li, B.; Zheng, C.; Wang, G. Molecular detection of fungal communities in the Hawaiian marine sponges *Suberites zeteki* and *Mycale armata*. *Appl. Environ. Microbiol.* **2008**, *74*, 6091-6101

Grossart, H.P.; Van den Wyngaert, S.; Kagami, M.; Wurzbacher, C.; Cunliffe, M.; Rojas-Jimenez, K. Fungi in aquatic ecosystems. *Nat. Rev. Microbiol.* **2019**, *17*, 339-354.

Hassett, B.T.; Vonnahme, T.R.; Peng, X.; Jones, E.B.G.; Heuze, C. Global diversity and geography of planktonic marine fungi. *Bot. Mar.* **2020**, *63*, 121-139.

Henriquez, M.; Vergara, K.; Norambuena, J.; Beiza, A.; Maza, F.; Ubilla, P.; Araya, I.; Chavez, R.; San-Martin, A.; Darias, J.; et al. Diversity of cultivable fungi associated with Antarctic marine sponges and screening for their antimicrobial, antitumoral and antioxidant potential. *World J. Microbiol. Biotechnol.* **2014**, *30*, 65-76.

Höoller, U.; Wright, A.D.; Matthee, G.F.; Konig, G.M.; Draeger, S.; Aust, H.J.; Schulz, B. Fungi from marine sponges: diversity, biological activity and secondary metabolites. *Mycol. Res.* **2000**, *104*, 1354-1365.

Jones, E.B.G.; Suetrong, S.; Sakayaroj, J.; Bahkali, A.H.; Abdel-Wahab, M.A.; Boekhout, T.; Pang, K.-L. Classification of marine Ascomycota, Basidiomycota, Blastocladiomycota and Chytridiomycota. *Fungal Divers.* **2015**, *73*, 1-72.

Kwon, S.L.; Park, M.S.; Jang, S.; Lee, Y.M.; Heo, Y.M.; Hong, J.H.; Lee, H.; Jang, Y.; Park, J.H.; Kim, C.; et al. The genus *Arthrinium* (Ascomycota, Sordariomycetes, Apiosporaceae) from marine habitats from Korea, with eight new species. *IMA Fungus* **2021**, *12*, 1-26.

Kutty, S.; Philip, R. Marine yeasts - A review. *Yeast* **2008**, *25*, 465-483.

Lai, X.; Cao, L.; Tan, H.; Fang, S.; Huang, Y.; Zhou, S. Fungal communities from methane hydrate-bearing deep-sea marine sediments in South China Sea. *ISME J* **2007**, *1*, 756-762.

Li C.L. Diversity of marine-derived fungi from seaweeds in intertidal zone of Qingdao and sediments of Bohai Sea. Master dissertation, Ocean University of China, **2013**.

Li, W.; Wang, M.; Bian, X.; Guo, J.; Cai, L. A high-level fungal diversity in the intertidal sediment of Chinese seas presents the spatial variation of community composition. *Front. Microbiol.* **2016**, *7*, 2098.

Li, D.; Wang, F.; Cai, S.; Zeng, X.; Xiao, X.; Gu, Q.; Zhu, W. Two new bisorbicillinoids isolated from a deep-sea fungus, *Phialocephala* sp. FL30r. *J. Antibiot.* **2007**, *60*, 317–320.

Li, L.; Singh, P.; Liu, Y.; Pan, S.; Wang, G. Diversity and biochemical features of culturable fungi from the coastal waters of Southern China. *AMB Express* **2014**, *4*, 60.

Li, Q.; Wang, G. Diversity of fungal isolates from three Hawaiian marine sponges. *Microbiol. Res.* **2009**, *164*, 233-241.

Jones, E.B.G.; Pang, K.-L.; Abdel-Wahab, M.A.; Scholz, B.; Hyde, K.D.; Boekhout, T.; Ebel, R.; Rateb, M.E.; Henderson, L.; Sakayaroj, J.; et al. An online resource for marine fungi. *Fungal Divers.* **2019**, *96*, 347-433.

Kalenitchenko, D.; Fagervold, S.K.; Pruski, A.M.; Vétion, G.; Yücel, M.; Le Bris, N.; Galand, P.E. Temporal and spatial constraints on community assembly during

microbial colonization of wood in seawater. *ISME J.* **2015**, *9*, 2657-2670.

Mahanty, S.; Bakshi, M.; Ghosh, S.; Chatterjee, S.; Bhattacharyya, S.; Das, P.; Das, S.; Chaudhuri, P. Green synthesis of iron oxide nanoparticles mediated by filamentous fungi isolated from sundarban mangrove ecosystem, India. *BioNanoScience* **2019**, *9*: 637-651.

Martorell, M.M.; Lannert, M.; Matula, C.V.; Quartino, M.L.; de Figueroa, L.I.C.; Mac Cormack, W.P.; Ruberto, L.A.M. Studies toward the comprehension of fungal-macroalgae interaction in cold marine regions from a biotechnological perspective. *Fungal Biology* **2021**, *125*, 218-230.

Maamar, A.; Lucchesi, M.-E.; Debaets, S.; van Long, N.N.; Quemener, M.; Coton, E.; Bouderbala, M.; Burgaud, G.; Matallah-Boutiba, A. Highlighting the crude oil bioremediation potential of marine fungi isolated from the Port of Oran (Algeria). *Diversity* **2020**, *12*, 196.

Miao, L.; Kwong, T.; Qian, P. Effect of culture conditions on mycelial growth, antibacterial activity, and metabolite profiles of the marine-derived fungus *Arthrinium* c.f. saccharicola. *Appl. Microbiol. Biotechnol.* **2006**, *72*, 1063-1073.

Nakagaki, K.; Hata, K.; Iwata, E.; Takeo, K. *Malassezia pachydermatis* isolated from a South American sea lion (Otaria byronia) with dermatitis. *J. Vet. Med. Sci.* **2000**, *62*, 901-903.

Neethu, C.S.; Saravanakumar, C.; Purvaja, R.; Robin, R.S.; Ramesh, R. Oil-Spill triggered shift in indigenous microbial structure and functional dynamics in different marine environmental matrices. *Sci. Rep.* **2019**, *9*, 1-13.

Orsi, W.; Biddle, J.F.; Edgcomb, V. Deep sequencing of subseafloor eukaryotic rRNA reveals active fungi across marine subsurface provinces. *PLoS One* **2013**, *8*, e56335.

Pham, T.T.; Dinh, K.V.; Nguyen, V.D. Biodiversity and Enzyme Activity of Marine Fungi with 28 New Records from the Tropical Coastal Ecosystems in Vietnam. *Mycobiology* **2021**, *49*, 559-581.

Panno, L.; Voyron, S.; Anastasi, A.; Mussat Sartor, R.; Varese, G. Biodiversity of marine fungi associated with the seagrass Posidonia oceanica: an ecological and biotechnological perspective. . *Biol. Mar. Mediterr.* **2011**, *18*, 85-88.

Passarini, M.R.Z.; Rodrigues, M.V.N.; da Silva, M.; Sette, L.D. Marine-derived filamentous fungi and their potential application for polycyclic aromatic hydrocarbon bioremediation. *Mar. Pollut. Bull.* **2011**, *62*, 364-370.

Raghukumar, S. Fungi in coastal and oceanic marine ecosystems. Springer: New York, USA, **2017**.

Rämä, T.; Nordén, J.; Davey, M.L.; Mathiassen, G.H.; Spatafora, J.W.; Kauserud, H. Fungi ahoy! Diversity on marine wooden substrata in the high North. *Fungal Ecol.* **2014**, *8*, 46-58.

Redou, V.; Ciobanu, M.C.; Pachiadaki, M.G.; Edgcomb, V.; Alain, K.; Barbier, G.; Burgaud, G. In-depth analyses of deep subsurface sediments using 454-pyrosequencing reveals a reservoir of buried fungal communities at record-breaking depths. *FEMS Microbiol. Ecol.* **2014**, *90*, 908-921.

Rédou, V.; Navarri, M.; Meslet-Cladière, L.; Barbier, G.; Burgaud, G. Species richness and adaptation of marine fungi from deep-subseafloor sediments. *Appl. Environ. Microbiol.* **2015**, *81*, 3571-3583.

Richards, T.A.; Jones, M.D.; Leonard, G.; Bass, D. Marine fungi: their ecology and molecular diversity. *Annu. Rev. Mar. Sci.* **2012,** *4*, 495-522.

Sakayaroj, J.; Preedanon, S.; Supaphon, O.; Jones, E.B.G.; Phongpaichit, S. Phylogenetic diversity of endophyte assemblages associated with the tropical seagrass *Enhalus acoroides* in Thailand. *Fungal Divers.* **2010**, *42*, 27-45.

Subrmaniyan, R.; Ponnambalam, S.; Thirunavukarassu, T. Inter species variations in cultivable endophytic fungal diversity among the tropical seagrasses. *Proc. Natl. Acad. Sci. India Sect. B Biol. Sci.* **2016**, *88*.

Sobolevskaya, M.P.; Berdyshev, D.V.; Zhuravleva, O.I.; Denisenko, V.A.; Dyshlovoy, S.A.; von Amsberg, G.; Khudyakova, Y.V.; Kirichuk, N.N.; Afiyatullov, S.S. Polyketides metabolites from the marine sediment-derived fungus *Thermomyces lanuginosus* Tsikl. KMM 4681. *Phytochemistry Lett.* **2021**, *41*, 114-118.

Steliga, T.; Jakubowicz, P.; Kapusta, P. Changes in toxicity during *in situ* bioremediation of weathered drill wastes contaminated with petroleum hydrocarbons. *Bioresour. Technol.* **2012**, *125*, 1-10.

Sen, K.; Sen, B.; Wang, G. Diversity, abundance, and ecological roles of planktonic fungi in marine environments. *J. Fungi.* **2022**, *8*, 491.

Sen, K.; Bai, M.; Sen, B.; Wang, G. Disentangling the structure and function of mycoplankton communities in the context of marine environmental heterogeneity. *Sci. Total Environ.* **2021**, *766*.

Singh, P.; Raghukumar, C.; Verma, P.; Shouche, Y. Phylogenetic diversity of culturable fungi from the deep-sea sediments of the Central Indian Basin and their growth characteristics. *Fungal Divers.* **2010**, *40*, 89-102.

Silber, J.; Kramer, A.; Labes, A.; Tasdemir, D. From discovery to production: Biotechnology of marine fungi for the production of new antibiotics. *Mar. Drugs* **2016**, *14*, 137.

Taha, H.; Shivanand, P.; Zainudin, M.; Hadanan, N. Identification of culturable marine fungi and bacteria from coastal region in Brunei Darussalam. *Biodiversitas* **2021**, *22*, 1326-1331.

Takishita, K.; Tsuchiya, M.; Reimer, J.; Maruyama, T. Molecular evidence demonstrating the basidiomycetous fungus *Cryptococcus curvatus* is the dominant microbial eukaryote in sediment at the Kuroshima Knoll methane seep. *Extremophiles* **2006**, *10*, 165-169.

Taylor, J.D.; Cunliffe, M. High-throughput sequencing reveals neustonic and planktonic microbial eukaryote diversity in coastal waters. *J. Phycol.* **2014**, *50*, 960-965.

Venkatachalam, A.; Thirunavukkarasu, N.; Suryanarayanan, T.S. Distribution and diversity of endophytes in seagrasses. *Fungal Ecol.* **2015**, *13*, 60-65.

Vera, J.; Gutiérrez, M.H.; Palfner, G.; Pantoja, S. Diversity of culturable filamentous Ascomycetes in the eastern South Pacific Ocean off Chile. World *J. Microbiol. Biotechnol.* **2017**, *33*, 1-13.

Wang, J.; Wang, Z.; Ju, Z.; Wan, J.; Liao, S.; Lin, X.; Zhang, T.; Zhou, X.; Chen, H.; Tu, Z.; et al. Cytotoxic cytochalasins from marine-derived fungus *Arthrinium*

arundinis. *Planta Med.* **2015**, *81*, 160-166.
Wang, M.; Ma, Y.; Cai, L.; et al. Seasonal dynamics of mycoplankton in the Yellow Sea reflect the combined effect of riverine inputs and hydrographic conditions. *Mol. Ecol*. **2021**, 3*0*, 3624-3637.
Xu, W.; Guo, S.; Pang, K.-L.; Luo, Z.-H. Fungi associated with chimney and sulfide samples from a south mid-atlantic ridge hydrothermal site: distribution, diversity and abundance. *Deep Sea Res. I Oceanogr. Res. Pap.* **2017**, *123*, 48-55.
Xu, W.; Luo, Z.H.; Guo, S.; Pang, K.L. Fungal community analysis in the deep-sea sediments of the Pacific Ocean assessed by comparison of ITS, 18S and 28S ribosomal DNA regions. *Deep Sea Res. I Oceanogr. Res. Pap.* **2016**, *109*, 51-60.
Zhang, Z.; He, X.; Che, Q.; Zhang, G.; Zhu, T.; Gu, Q.; Li, D. Sorbicillasins A–B and Scirpyrone K from a Deep-Sea-Derived Fungus, *Phialocephala* sp. FL30r. *Mar. Drugs* **2018**, *16*, 245.
Zhang, T.; FeiWang, N.; Qin Zhang, Y.; Yu Liu, H.; Yan Yu, L. Diversity and distribution of fungal communities in the marine sediments of Kongsfjorden, Svalbard (High Arctic). *Sci. Rep.* **2015**, *5*, 14524.
Zhang, X.Y.; Tang, G.L.; Xu, X.Y.; Nong, X.H.; Qi, S.H. Insights into deep-sea sediment fungal communities from the east indian ocean using targeted environmental sequencing combined with traditional cultivation. *PLoS ONE* **2014**, *9*, e109118.
Zhdanova, N.N.; Zakharchenko, V.A.; Vember, V.V.; Nakonechnaya, L.T. Fungi from Chernobyl: mycobiota of the inner regions of the containment structures of the damaged nuclear reactor. *Mycol. Res.* **2000**, *104*, 1421-1426.
Zuccaro, A.; Summerbell, R.C.; Gams, W.; Schroers, H.J.; Mitchell, J.I. A new *Acremonium* species associated with *Fucus* spp., and its affinity with a phylogenetically distinct marine *Emericellopsis* clade. *Stud. Mycol.* **2004**, 283-297.
