# Supplementary material for: Different Geographic Strains of Dinoflagellate Karlodinium veneficum Host Highly Diverse Fungal Community and Potentially Serve as Possible Niche for Colonization of Fungal Endophytes
Source: Int J Mol Sci. 2023 Jan 14;24(2):1672. doi: 10.3390/ijms24021672 (PMC9865425; doi:10.3390/ijms24021672)
Supplement: Supplementary file 1 [file ijms-24-01672-s001.zip › Supplementary Table S6.pdf]

Table S6. Published records of 15 abundant genera in original/adjacent seawaters in which 13 *Karlodinium veneficum* cultures were initially established (Note that this table is not an exhaustive list but rather a list of publications represents a portion documenting the presence of these genera concerned). A total of 13 strains of *K. veneficum* originally isolated from 5 geographic origins were employed in this study, 12 of which were from coastal waters of China and one from Chesapeake Bay, USA. Among them 7 strains (KV6, KV8, KV10, KV11, KV12, KV13 and KV15) were from the Yellow Sea (Jiaozhou Bay, Shandong Province), one (KV7) from the Bohai Sea (Dongying, Shandong Province), two (KV1 and KV9) from the East China Sea (Ningde, Fujian Province), two (KV16 and KV17) from the South China Sea (Pearl River Estuary, Guangdong Province and Beibu Gulf, Guangxi Province). The strain KV4 was isolated from Chesapeake Bay in the USA (see Table 2 in the main text for details).

Genus	The strains where it presents in this study	Published records in the adjacent seawaters where the 13 cultures were initially established
Thyridium[1]	All the 13 samples	South China Sea (Li et al., 2016); East China Sea (Li et al., 2016); Bohai Sea (Li et al., 2016); Yellow Sea (Li, 2013; Wang et al., 2021)
Pseudeurotium	All the 13 samples	South China Sea (Li et al., 2016); East China Sea (Li et al., 2016); Bohai Sea (Li, 2013, marine sediments; Li et al., 2016); Yellow Sea (Li et al., 2016; Wang et al., 2021)
Candida	All the 13 samples	Pearl River Estuary, South China Sea (Li et al., 2014); South China Sea (Sun et al., 2017; Wang et al., 2019; Sen et al., 2021); East China Sea (Li et al., 2016); Bohai Sea (Li et al., 2016); Yellow Sea (Sun, 2011; Li, 2013; Wang et al., 2021); Chesapeake Bay, USA (Walker et al., 1975; Motta, 1978)

Cladosporium	KV1 (Ningde, East China Sea); KV4 (Chesapeake Bay in USA); KV8 (Jiaozhou Bay, Yellow Sea); KV9 (Ningde, East China Sea); KV10 (Jiaozhou Bay, Yellow Sea); KV12 (Jiaozhou Bay, Yellow Sea); KV16 (Pearl River Estuary, South China Sea); KV17 (Beibu Gulf, South China Sea)	Pearl River Estuary, South China Sea (Li et al., 2014); South China Sea (Wang et al., 2019; Sen et al., 2021); East China Sea (Feng, 2012, marine sediments; Li et al., 2016); Yellow Sea (Sun, 2011; Feng, 2012, marine sediments; Li, 2013; Wang et al., 2021); Sargasso Sea, USA (Duan et al., 2021); Chesapeake Bay, USA (Walker et al., 1975; Motta, 1978; Newell, 1981)
UC Ascomycota	KV7 (Dongying, Bohai Sea), KV9 (Ningde, East China Sea); KV10 (Jiaozhou Bay, Yellow Sea); KV11 (Jiaozhou Bay, Yellow Sea); KV12 (Jiaozhou Bay, Yellow Sea); KV13 (Jiaozhou Bay, Yellow Sea); KV15 (Jiaozhou Bay, Yellow Sea); KV16 (Pearl River Estuary, South China Sea); KV17 (Beibu Gulf, South China Sea)	-
Arthrinium	KV1 (Ningde, East China Sea); KV8 (Jiaozhou Bay, Yellow Sea); KV9 (Ningde, East China Sea); KV10 (Jiaozhou Bay, Yellow Sea); KV11 (Jiaozhou Bay, Yellow Sea); KV16 (Pearl River Estuary, South China Sea)	Pearl River Estuary, South China Sea (Li et al., 2014); South China Sea (Li et al., 2016; Wang et al., 2019); East China Sea (Li et al., 2016); Bohai Sea (Li, 2013, marine sediments); Yellow Sea (Sun, 2011; Li, 2013; Wang et al., 2021)
Tetracladium	KV4 (Chesapeake Bay in USA); KV8 (Jiaozhou Bay, Yellow Sea); KV15 (Jiaozhou Bay, Yellow Sea); KV16 (Pearl River Estuary, South China Sea); KV17 (Beibu Gulf, South China Sea)	South China Sea (Li et al., 2016); Yellow Sea (Wang et al., 2021)

Cryptococcus	KV1 (Ningde, East China Sea); KV4 (Chesapeake Bay in USA); KV9 (Ningde, East China Sea); KV10 (Jiaozhou Bay, Yellow Sea); KV11 (Jiaozhou Bay, Yellow Sea); KV12 (Jiaozhou Bay, Yellow Sea); KV13 (Jiaozhou Bay, Yellow Sea); KV17 (Beibu Gulf, South China Sea)	South China Sea (Li et al., 2016); East China Sea (Li et al., 2016); Yellow Sea (Wang et al., 2021)
Malassezia	KV1 (Ningde, East China Sea); KV4 (Chesapeake Bay in USA); KV6 (Jiaozhou Bay, Yellow Sea); KV7 (Dongying, Bohai Sea); KV9 (Ningde, East China Sea); KV10 (Jiaozhou Bay, Yellow Sea); KV11(Jiaozhou Bay, Yellow Sea); KV17 (Beibu Gulf, South China Sea)	South China Sea (Sun et al., 2017; Wang et al., 2019; Sen et al., 2021); East China Sea (Li et al., 2016); Bohai Sea (Wang et al., 2018); Yellow Sea (Wang et al., 2021); Sargasso Sea, USA (Duan et al., 2021)
Fusarium	KV1 (Ningde, East China Sea); KV7 (Dongying, Bohai Sea); KV12 (Jiaozhou Bay, Yellow Sea); KV13 (Jiaozhou Bay, Yellow Sea); KV15 (Jiaozhou Bay, Yellow Sea); KV17 (Beibu Gulf, South China Sea)	South China Sea (Wang et al., 2019; Sen et al., 2021); East China Sea (Feng, 2012, marine sediments; Li et al., 2016); Bohai Sea (Li, 2013, marine sediments; Li et al., 2016); Yellow Sea (Sun, 2011; Feng, 2012, marine sediments; Li, 2013; Wang et al., 2021); Chesapeake Bay, USA (Motta, 1978)
Acremonium	KV1 (Ningde, East China Sea); KV4 (Chesapeake Bay in USA); KV9 (Ningde, East China Sea); KV10 (Jiaozhou Bay, Yellow Sea); KV16 (Pearl River Estuary, South China Sea); KV17 (Beibu Gulf, South China Sea)	South China Sea (Li et al., 2016); East China Sea (Feng, 2012, marine sediments; Li et al., 2016); Bohai Sea (Li, 2013, marine sediments); Yellow Sea (Sun, 2011; Feng, 2012, marine sediments; Li, 2013; Wang et al., 2021); Chesapeake Bay, USA (Newell, 1981)

UC Fungi	KV1 (Ningde, East China Sea); KV4 (Chesapeake Bay in USA); KV13 (Jiaozhou Bay, Yellow Sea); KV15 (Jiaozhou Bay, Yellow Sea); KV17 (Beibu Gulf, South China Sea)	-
Phialocephala	KV13 (Jiaozhou Bay, Yellow Sea); KV15 (Jiaozhou Bay, Yellow Sea)	Yellow Sea (Li et al., 2016; Wang et al., 2021)
Teratosphaeria	KV12 (Jiaozhou Bay, Yellow Sea); KV13 (Jiaozhou Bay, Yellow Sea); KV15 (Jiaozhou Bay, Yellow Sea)	South China Sea (Sen et al., 2021); Yellow Sea (Li et al., 2016; Wang et al., 2021)
Thermomyces	KV8 (Jiaozhou Bay, Yellow Sea); KV9 (Ningde, East China Sea)	East China Sea (Li et al., 2016); Yellow Sea (Wang et al., 2021)

UC: unclassified.

[1] *Thyridium*: formerly as *Phialemoniopsis* (Sugita and Tanaka, 2022) and *Phialemonium* (Perdomo et al. 2013).

Sugita, R.; Tanaka, K. *Thyridium* revised: Synonymisation of *Phialemoniopsis* under *Thyridium* and establishment of a new order, Thyridiales. *MycoKeys* 2022, *86*, 147.

Perdomo, H;; Garcia, D.; Gene, J.; Cano, J.; Sutton, D.A.; Summerbell, R.; Guarro, J. *Phialemoniopsis*, a new genus of Sordariomycetes, and new species of *Phialemonium* and *Lecythophora*. *Mycologia* 2013, *105*, 398-421.

References

Duan, Y.; Xie, N.; Wang, Z.; Johnson, Z.I.; Hunt, D.E.; Wang, G. Patchy distributions and distinct niche partitioning of mycoplankton populations across a nearshore to open ocean gradient. *Microbiology spectrum*, **2021**, *9*, e01470-21.

Feng C.H. The primary study on the diversity of fungi from sediment from Yellow Sea and Yangtze Estuary. Master dissertation, Ocean University of China, **2012**.

Li C.L. Diversity of marine-derived fungi from seaweeds in intertidal zone of Qingdao and sediments of Bohai Sea. Master dissertation, Ocean University of China, **2013**.

Li, L.; Singh, P.; Liu, Y.; Pan, S.; Wang, G. Diversity and biochemical features of culturable fungi from the coastal waters of Southern China. *Amb Express.* **2014**, *4*, 1-11.

Li, W.; Wang, M.; Bian, X.; Guo, J.; Cai, L. A high-level fungal diversity in the intertidal sediment of Chinese seas presents the spatial variation of community composition. *Front. Microbiol.* **2016**, *7*, 2098.

Motta, J.J. The occurrence of fungi on some rooted aquatics from the Chesapeake Bay. *Estuaries* **1978**, *1*, 101-105.

Newell, S.Y. Fungi and bacteria in or on leaves of eelgrass (*Zostera marina* L.) from Chesapeake Bay. *Applied and Environmental Microbiology* **1981**, 41, 1219-1224.

Sen, K.; Bai, M.; Sen, B.; Wang, G. Disentangling the structure and function of mycoplankton communities in the context of marine environmental heterogeneity. *Sci. Total Environ.* **2021**, *766*, 142635.

Sun, J.Y.; Song, Y.; Ma, Z.P.; Zhang, H.J.; Yang, Z.D.; Cai, Z.H.; Zhou, J. Fungal community dynamics during a marine dinoflagellate (*Noctiluca scintillans*) bloom. *Mar. Environ. Res.* **2017**, *131*, 183-194.

Sun, K.M. The primary study on the diversity of marine-derived fungi associated with marine algae and sediment from the intertidal zone of Shandong, Yellow Sea. Master dissertation, Ocean University of China, **2011**.

Wang, Y.; Sen, B.; He, Y.; Xie, N.; Wang, G. Spatiotemporal distribution and assemblages of planktonic fungi in the coastal Waters of the Bohai Sea. *Front. Microbiol.* **2018**, *9*, 584

Wang, Y.; Sen, K.; He, Y.; Xie, Y.; Wang, G. Impact of environmental gradients on the abundance and diversity of planktonic fungi across coastal habitats of contrasting trophic status. *Sci. Total Environ.* **2019**, *683*, 822-833.

Wang, M.; Ma, Y.; Cai, L.; et al. Seasonal dynamics of mycoplankton in the Yellow Sea reflect the combined effect of riverine inputs and hydrographic conditions. *Mol. Ecol*. **2021**, 3*0*, 3624-3637.

Walker, J.D.; Austin, H.F.; Colwell, R.R. Utilization of mixed hydrocarbon substrate by petroleum-degrading microorganisms. *The Journal of General and Applied Microbiology* **1975**, *21*, 27-39.
