# Supplementary material for: Different Geographic Strains of Dinoflagellate Karlodinium veneficum Host Highly Diverse Fungal Community and Potentially Serve as Possible Niche for Colonization of Fungal Endophytes
Source: Int J Mol Sci. 2023 Jan 14;24(2):1672. doi: 10.3390/ijms24021672 (PMC9865425; doi:10.3390/ijms24021672)
Supplement: Supplementary file 1 [file ijms-24-01672-s001.zip › Supplementary Table S7.pdf]

**Table S7. Control information from other 5 cultures of microalgae examined for fungal presence with same high-throughput gene amplicon sequencing targeted at rDNA ITS region.** All these 5 cultures and the 13 *Karlodinium veneficum* cultures examined in this study were cultivated and processed in the same protocols in our laboratory.

| Samples | Genus/species | Origin/ Isolation date | Accession No.[1] | *Thyridium* [2] | *Pseudeurotium* | *Candida* |
|---|---|---|---|---|---|---|
| KD | *Karlodinium decipens* | Jiaozhou Bay, Shandong Province (Yellow Sea, China), 2019 | PRJNA892752 | Y[3] | N | N |
| PF | *Pseliodinium fusus* | Jiaozhou Bay, Shandong Province (Yellow Sea, China), 2019 | PRJNA892740 | N | N | Y |
| BB | *Biecheleria brevisulcata* | Ningde, Fujian Province (East China Sea), 2018 | PRJNA892739 | N | N | N |
| AA | *Aureococcus anophagefferens* | USA, 1986 [4] | PRJNA892281 | Y | N | Y |
| AS | *Akashiwo sanguinea* | Northport Bay, New York, USA, 2011 | PRJNA892755 | N | N | N |

[1] The accession number of the high-throughput gene amplicon sequencing data for rDNA ITS region of the sample in the NCBI Short Read Archive (SRA) database.

[2] *Thyridium*: formerly as *Phialemoniopsis* (Sugita and Tanaka, 2022, MycoKeys, 86, 147) and *Phialemonium* (Perdomo et al. 2013, Mycologia, 105, 398-421).

[3] Y/N: the fungal genus has been/ has not been detected in the sample.

[4] The culture of *Aureococcus anophagefferens* strain CCMP1984 (CCMP, Culture Collection of Marine Phytoplankton) was obtained from Stony Brook University and cultured in our laboratory since 2013.
